# Progress in State Medicine

**Published:** 1898-06-11

**Authors:** 


### Progress in State Medicine.

### PUBLIC HEALTH.

**Sewage and Refuse Disposal.**—Tichborne[1] discusses the methods of dissemination of the micro-organisms found in sewer air. Such organisms gain access not only by the bursting of gas bubbles in the sewage, but by violent concussions breaking the sewage into spray. The condensed vapour carrying the germs is capable of travelling through the whole system of drainage, may be drawn into a warm shaft connected with a dwelling-house, where the vapour is converted into a permanent gas, and the germs liberated. Enteric fever, diarrhœa, and diphtheria are the diseases most commonly associated with sanitary defects. The very large quantities of chemicals required, and the consequent expense, have proved a bar to all attempts at sewage disinfection. By rubbing together crystallised phenol one part, camphor three parts, naphthaline one-half part, a fluid is obtained of very high bactericidal powers. Being of low specific gravity, this fluid, when mixed with sewage, forms a fine pellicle on the surface, and all fluids that are volatilised or mechanically eliminated by the escape of gas must pass through the germicide layer. At the same time the carbolic acid slowly diffuses through the mass, so that not only is the air protected from pollution, but the sewage slowly undergoes disinfection. Instead of the expensive mixture above mentioned "light coal tar oil" may be substituted, and is equally efficacious.

The safe disposal of typhoid dejecta is a matter of much difficulty. In water-closet towns, stools, if not disinfected, may specifically contaminate the sewers and watercourses. Strong disinfectants added to stools coagulate albuminous matter and thus protect the bacillus with an insoluble casing. [2]A good method of dealing with the dejecta is to line the bed-pan with a layer of some absorbent material, such as peat-moss, cotton-wool, or sawdust, so that eventually the contents may be destroyed by burning in a bright fire in a kitchen range. The bed-pan is then filled with perchloride solution, and left for a couple of hours. In big institutions, fever hospitals, &c., the dejecta are best treated by the "Newcastle Steriliser," a hermetically closed iron vessel of 30 gallons capacity, in which they are boiled under steam pressure. In towns where the pail system of night soil removal is in use, a special coloured pail, with an air-tight lid, and charged with some strong disinfectant, is sent to the houses where typhoid fever is known to exist. This pail is removed at short intervals, and its contents sent to the destructor. It is in midden towns more especially that a difficulty arises; indeed, it is practically impossible to ensure the disinfection of a midden once polluted by typhoid dejecta, and, as these receptacles are seldom impervious, the surrounding soil soon becomes saturated with infective fæcal matter. Scurfield[3] has reported on the connection between middens and typhoid fever. Comparing various large towns, he finds that, whilst in midden towns 26 persons per 100,000 of the population die annually from typhoid fever, only 15 per 100,000 die from the same disorder in water-closet towns. Taking the case of Nottingham, where both systems are at work, he finds that in a given ten years one case of typhoid fever occurs per 120 houses with pails, one case per 558 houses with water-closets, and one case per 37 houses with privies. Robertson, medical officer of health for Sheffield, examined the soil around infected privies, but failed to find the bacillus typhosus. His further experiments go to prove, however, that the bacillus can both live and multiply in normal soil if supplied with organic nutriment, and that although sunlight and the presence of vegetation are prejudicial to its welfare, yet the organism may survive in the soil for a period of twelve months. Dr. Sidney Martin,[4] using organically polluted soils taken from localities where enteric fever was endemic, found that the bacillus typhosus multiplied and spread under favourable conditions of moisture and temperature, and were still living at the end of a period of 268 days. In virgin, peaty, and red sandy soils the organisms showed no signs of vitality at the end of 14 days. The factors favouring the growth of the bacillus typhosus in soil are organic pollution, moisture, and warmth. To prevent organic pollution every effort must be made to keep pure the soil about our houses by diligent sweeping and scavenging, and by the abolition of the privy-midden. The moisture and temperature of the soil is in towns (where the influence of sunlight and alternating weather is least felt) best regulated by the systematic paving of house yards with impervious material, and by an efficient system of drainage and sewerage.

The attempts made up to the present to devise a means for the immediate destruction of fæces by fire have not been successful. The apparatus of Sheiding and Linholdt has not been found workable, and the plant in use at Newcastle for the manufacture of poudrette is intended to deal with the sewage of a large town. [5]Recently at Redlitz artillery barracks a "fire closet" has been erected and most successfully worked. From a long trough the fæcal matter runs to a finely-meshed grating, where the urine strains off. When a sufficient bulk of these materials has collected a furnace is lighted, evaporates the urine, and dries and partially destroys and incinerates the solid material. The unburned gases, smoke, and dust are drawn into a second furnace, where their destruction is then completed. The furnaces are of such a size as to be able to deal with the excrement of 350 to 500 persons, and are worked for ten hours every fourth day. Oil or coke may be used as fuel, small quantities only being required, for the material operated upon contains 23 per cent. of combustible matter. The ash amounts to three grammes per head per day, and may be used for manure or for road making. The fire-closet may be utilised for liquids only, and is equally efficacious. Where water-carriage or sewage farming is impossible it may prove of great service.

One of the strongest objections to earth-closets has been the fact that bacilli may remain alive for almost indefinite periods in mould or ashes. Sinnhuler,[6] however, states that if 10 per cent. of quicklime be added to dry mould it acquires bactericidal powers, and retains them for a long period. With the addition of 3 per cent. of quicklime, cholera vitrios died in four hours, and with 8 per cent. addition they died in 15 minutes.

Petter[7] has devised an ingenious domestic refuse-destructor for disposing of such materials as potato-peelings, cabbage leaves, fish bones, &c. The destructor is part of the kitchen range, the ashpit of which is completely enclosed for drying the material. Any grease or fat is absorbed by the ash falling through the fire-bars. When thoroughly dried the waste material is shovelled into the fire, the draught for which is provided by means of holes in the door. All fumes are destroyed by being carried through the fire. The range is really a slow combustion stove, and, being also partly fed by domestic refuse, is very economical in its working. Its adoption would do much to improve the present offensive condition of household ashpits or dustbins.

[1] Dublin Jour. Med. Sci., July, 1897. [2] Brit. Med. Jour., Nov. 13. [3] Rep. to Corp. of Sunderland. [4] Local Gov. Bd. Rep., 1896-7. [5] Jour. d'Hygiene, July 8. [6] Inaug. Dissert. Konigsb., 1896. [7] Sanit. Rec., Feb. 25.

